# Safety and efficacy of liposomal amphotericin B for treatment of complicated visceral leishmaniasis in patients without HIV, North-West Ethiopia

**DOI:** 10.1186/s12879-016-1746-1

**Published:** 2016-10-10

**Authors:** Aschalew Tamiru, Bethlehem Tigabu, Sisay Yifru, Ermias Diro, Asrat Hailu

**Affiliations:** 1 Leishmaniasis Research and Treatment Center, University of Gondar, College of Medicine and Health Science, Gondar, Ethiopia; 2 Tuberculosis Clinic, University of Gondar, College of Medicine and Health Science, Gondar, Ethiopia; 3 Department of Pediatrics, University of Gondar, College of Medicine and Health Science, Gondar, Ethiopia; 4 Department of Internal Medicine, University of Gondar, College of Medicine and Health Science, Gondar, Ethiopia; 5 Department of Microbiology, Immunology and Parasitology, College of Health Sciences, Addis Ababa University, Addis Ababa, Ethiopia

**Keywords:** Liposomal amphotericin B, Visceral leishmaniasis, Safety, Efficacy

## Abstract

**Background:**

Visceral leishmaniasis (VL) is a protozoan disease that is invariably fatal if left untreated. The disease is found in 70 countries with incidence of 0.2 – 0.4 million cases. The mainstay of treatment in resource limited countries like Ethiopia is antimonials, while use of liposomal amphotericin B is reserved for treatment of complicated VL cases. The aim of this study was to assess the safety and efficacy of liposomal amphotericin B in HIV negative VL patients diagnosed with complications.

**Methods:**

A retrospective chart review was conducted involving records of patients admitted between January 2009 and December 2014. Baseline sociodemographic, clinical, and treatment outcome data were collected. The doses of liposomal amphotericin B and adverse events related to treatment were retrieved. Categorical and continuous variables respectively were analyzed by Chi-square and Mann–Whitney U tests. A *p*-value of less than 0.05 was considered statistically significant.

**Results:**

A total of 147 patients with severe VL were treated with liposomal amphotericin B in total dose ranges of 20 mg/kg to 35 mg/kg. In the overall treatment outcome analysis, initial cure (30 days after start of treatment) was observed in 128 (87.1 %), treatment failures in 10 (6.8 %), interruptions in 2(1.4 %) and deaths in 7 (4.8 %) patients. Initial cure rate at high dose (24-35 mg/kg total dose) was 96.7 % (59/61) versus 80.2 % (69/86) at lower doses (<24 mg/kg); which was significantly higher (*P* < 0.01), OR = 4.56: 95 %, Confidence Interval (CI) = 1.17 – 20.78). Ten cases (11.8 %) of treatment failure occurred in the low dose treatment group. The most common adverse events (AEs) were hypokalemia in 39 cases (26.5 %) and infusion related reactions in 16 (10.9 %). The frequency of hypokalemia and infusion related reactions were not significantly different between the low and high dose liposomal amphotericin B.

**Conclusion:**

In HIV negative complicated VL patients, high dose of liposomal amphotericin B was found to have high cure rate at the end of treatment. The appropriate dose for better efficacy needs to be determined. Monitoring serum potassium level during treatment with liposomal amphotericin B should be an essential component of the clinical management of VL.

## Background

Visceral leishmaniasis (VL), also called kala-azar, is a protozoan parasitic neglected disease that is transmitted by sand flies belonging to the genera Phlebothomus and Lutzomyia. The disease is a worldwide health problem affecting over 70 countries with annual incidence of 0.2 – 0.4 million cases. India, Bangladesh, Sudan, South Sudan, Ethiopia and Brazil account for the majority of cases [1]. It is caused by *Leishmania donovani* (Asia and Africa), or *Leishmania infantum* (Southern Europe and South America). The disease is characterized by fever, splenomegaly, anemia, weight loss, abdominal swelling, bleeding, and pancytopenia. Peripheral lymphadenopathy is common in some foci [2]. If left untreated, the disease is universally fatal.

VL is a significant public health problem in Ethiopia. Its incidence is estimated to be 3,700 – 7,400 per year [3]. The northwestern regions of Ethiopia accounts for about 60 % of the case load in Ethiopia. In this region of Ethiopia, young migrant workers are the most affected [4].

There are limited drugs available to treat VL. The currently used anti-leishmanial drugs are antimonials (sodium stibogluconate [SSG] and meglumine antimoniate [glucantime]), liposomal amphotericin B, paromomycin and miltefosine. Currently, the standard treatment in East Africa is a combination of SSG and paromomycin for 17 days. While SSG has been the mainstay of treatment for VL since 1940s, resistance has become a concern in the Indian subcontinent [5, 6].

Liposomal amphotericin B (Gilead Sciences) was first used in humans for the treatment of multidrug resistant Mediterranean VL in 1990 [7]. Since then, the drug has increasingly been used in endemic regions. Short-course regimens give cure rates of more than 90 % in the Indian subcontinent [8]. It also has good safety profile. It causes minor adverse events, commonly infusion related reactions (fever, chills, arthralgia) and rarely renal toxicity. Encouraged by its high efficacy and low toxicity, the World Health Organization (WHO) endorsed liposomal amphotericin B as a drug with the highest therapeutic index of existing anti-leishmanial dugs [9]. Nevertheless, treatment outcomes vary by geographic locations. While there was good treatment response in India, it was less efficacious and inconsistent in Brazil and East Africa [10].

There is limited experience in use of liposomal amphotericin B in East Africa due to cost implications. It was first introduced to Ethiopia in 2006 as first-line treatment for HIV-positive and severely ill immunocompetent VL patients [11]. Since then its use is primarily limited to patients with severe disease and HIV co-infection. The experience from Médecins Sans Frontière (MSF) programs in Sudan showed lower cure rate at a total dose of 20 mg/kg [12].

A study in Ethiopia involving 94 HIV negative severely ill VL patients showed 93 % initial cure and 6 % death. However, among 195 VL-HIV co-infected patients, the initial cure rate was only 60 %, with 7 % deaths, and 32 % parasitological failure [11]. Liposomal amphotericin B was even less effective in treating HIV-positive VL relapses with reports of 38 % initial cure and 56 % parasitological failure [13]. Higher doses are now recommended to treat VL in HIV positive patients [14].

In the past, low dose liposomal amphotericin B (21 mg/kg) had been in use in Ethiopia for non-HIV VL patients; but assessments of treatment outcomes from time to time were not done. In this study, we aimed to study the treatment outcomes of liposomal amphotericin B in VL patients treated under routine settings.

## Methods

### Study design

Medical records (Charts) of VL patients treated with liposomal amphotericin B were reviewed retrospectively between Jan/2009 and Dec/2014 at the University of Gondar Leishmaniaisis Research and Treatment Center (LRTC).

### Study setting

LRTC is a clinical trial center established in 2004 in collaboration with Drugs for Neglected Diseases initiatives (DNDi). In this centre, several clinical trials have been conducted. The staff at LRTC are trained and experienced in Good Clinical Practice (GCP). The center has its own lab, pharmacy and chart archiving rooms. VL suspected patients are referred from the different units of the Hospital in Gondar and from other health facilities of the catchment area. Patients admitted for VL treatment in LRTC are routinely evaluated and the findings are documented in their chart records. Weekly follow up assessment with hematology, blood chemistry and electrolyte measurements are a routine practice in the treatment center.

### Diagnosis

Diagnosis of VL was carried out by microscopic demonstration of LD bodies in tissue aspirates and/or a positive rK39 serology test in a patient who fulfills the clinical case definition of VL (fever of greater than two weeks, and splenomegaly and/or lymphadenopathy with either loss of weight or anemia).

### Treatment protocol

Liposomal amphotericin B is used as an alternative therapy in case of failure of treatment with first-line drugs and in complicated VL patients; which includes those with HIV co-infection, edematous malnutrition, and deranged liver and renal function tests. The commonly used dosage is 3–5 milligram per kilogram in six to eight divided doses. Additional doses may be given when there is partial response to achieve complete cure. Doses are usually approximated to minimize wastage of left over medication in the last vial. Each dose is administered as intravenous infusion in 500 ml of 5 % dextrose in water solution over 2 h.

For this study, we classified the dosages of liposomal amphotericin B used as “high dose” for total dosage ranging from 24 – 35 mg/kg or “low dose” when it was less than 24 mg/kg. Treatment outcome was assessed at the end of one month of treatment. Those patients who were not cured at end of treatment were re-treated either with liposomal amphotericin B or SSG. All patients were also followed for possible adverse events. The treatment practice maintained an evaluation of treatment outcomes at three and six months following treatment.

### Data collection and management

A standardized data collection tool was prepared to capture the important data for the study. Sociodemographic, clinical, and laboratory results were retrieved from the treatment chart records; which included sex, age and residence, and clinical manifestations such as fever, splenomegaly and weight loss. The laboratory data included total white blood cell counts, hemoglobin levels, platelet counts, blood chemistry and electrolyte analyses. Data on treatment monitoring, adverse events and outcome of treatment were also recorded. Charts of patients with HIV co-infection and those with incomplete data were excluded. The collected data was entered on excel spreadsheet, checked for completeness and transferred to SPSS version 16.0 for analysis.

### Data analysis

Descriptive analysis of the socio-demographic data and clinical manifestations was carried out to obtain mean and median values with their standard deviation (SD) and interquartile range (IQR) respectively. Efficacy was assessed as clinical and/or parasitological cure at the end of one month treatment (initial cure). Test of cure (ToC) for assuring parasitological cure was done by tissue aspiration only when there is persistence of some VL signs and symptoms. Clinical cure was defined as regression of splenomegaly and abatement of fever, and by clinical improvement (weight gain, recovery in hematological indices). The frequencies of adverse events related to liposomal amphotericin B administration were calculated. Treatment outcomes were compared between different dosages. Categorical data were analyzed using chi-square and Fisher’s exact test as appropriate. Continuous variables were analyzed using Mann–Whitney *U* test. A *p*-value of less than 0.05 was considered statistically significant.

## Results

In the period January 2009 – December 2014, there were 147 non-HIV VL patient charts showing treatment with liposomal amphotericin B. The socio-demography and clinical presentations are summarized in Table 1. The median age of patients on admission was 25 years with an interquartile range (IQR) of 20–28. The majority (*n* = 144, 98 %) were males. The mean body mass index (BMI) was 16.7 kg/m^2^. Demographically, 128 (87 %) were migrant laborers; the rest were stable residents (*n* = 16, 8.3 %) or settlers (*n* = 3, 2.0 %).

**Table 1:** Sociodemographic and baseline characteristics of VL patients

Variables	Frequency	Percentage (%)
Age: Median (Interquartile range)	25 (20–28)	-
Sex		
Male	144	98
Female	3	2
Type of settlement		
Seasonal migrant workers	128	87
Stable residents	16	10.9
Settlers	3	2
Clinical features		
Fever:		
Yes	138	93.9
No	9	6.1
Weight loss		
Yes	138	93.9
No	9	6.1
Loss of appetite		
Yes	138	93.9
No	9	6.1
Abdominal swelling		
Yes	57	38.8
No	90	61.2
Splenomegaly		
Yes	138	93.9
No	9	6.1
Lymphadenopathy		
Yes	15	10.2
No	132	89.8
Method of diagnosis		
Parasitological	122	83
Clinical and RDT using rk-39	25	17
Laboratory and physical examination		
White blood cell count (WBC), [Median (IQR) x 10^3^ cells /μl]	1.5 (1.1 – 2.1)	-
Platelet counts: [median (IQR) x 10^3^ Cells/μl]	53 (34 – 89)	-
Spleen size, mean ± SEM (cm)	8.5 ± 0.38	-
Temperature, median (IQR) in °c	38.5 (38–39)	-
Body mass index: Mean ± SEM	16.8 ± 0.14	
Concomitant diseases/conditions		
CAP	17	11.6
Pulmonary TB	7	4.8
Malaria	11	7.5
Acute otitis media	3	2
Elevated creatinine at baseline	11	7.5
Other conditions	59	40.1

With regards to clinical features, the vast majority had history of fever (*n* = 138, 93.9 %). Loss of appetite and weight loss each occurred in 138 (93.9 %) patients. Other clinical features were abdominal swelling (*n* = 57, 38.8 %), splenomegaly (*n* = 138, 93.9 %), cough (*n* = 79, 53.7 %), diarrhea (*n* = 31, 21.1 %) and lymphadenopathy (*n* = 15, 10.2 %).

Diagnosis of VL was confirmed parasitologically in 122 (83 %), while in the remaining 25 (17 %) it was made by fulfilled clinical case definition and serologically by rk-39 RDT. Concomitant diseases such as community acquired pneumonia (CAP), pulmonary tuberculosis (PTB) and malaria were found in 17(11.6 %), 7 (4.8 %) and 11 (7.5 %) of patients respectively. Acute otitis media was seen in 3(2 %) patients. Elevated creatinine 11(7.5 %). Other conditions such as severe neutropenia and severe anemia were observed in 59 (40.1 %).

### Overall treatment outcomes and initial responses of VL patients treated with low and high dose liposomal amphotericin B

The median (and IQR) total dose of liposomal amphotericin B used per patient was 24 mg/kg (21 – 30 mg/kg). At the end of treatment, 76 (51.7 %) patients were declared clinically cured, while 63 (42.9 %) required a test of cure. The remaining 8 (5.4 %) patients died or defaulted before completing full treatment course. Combining clinical and parasitological assessment criteria, overall initial cure was achieved in 128 (87.1 %). The remaining were either treatment failures (*n* = 10, 6.8 %), defaulters (*n* = 2, 1.4 %), or deaths (*n* = 7, 4.8 %) [Table 2]. Comparison of initial treatment outcomes of high and low dose liposomal amphotericin B is summarized in Table 3. Among 86 patients who received low dose liposomal amphotericin B (<24 mg/kg total dose), the initial cure rate was 80.2 % (*n* = 69). The rest were either treatment failures (*n* = 10, 11.62 %), defaulters (*n* = 2, 2.3 %), or deaths (*n* = 5, 5.8 %). Among those treated with high dose liposomal amphotericin B (24-35 mg/kg total dose; *n* = 61), 59 (96.7 %) were cured at the end of treatment (initial cure), with two deaths and no treatment failure. The difference in the initial cure rate between those treated at high and low doses of liposomal amphotericin B was significant (*p* < 0.05) (96.7 % versus 80.2 %; OR 4.56; 95 % Confidence Interval (CI): 1.17 – 20.78).

**Table 2:** The overall initial treatment outcomes

Variable	Outcome	Frequency	Percent
Initial treatment outcome	Cured	128	87.1
	Treatment failure	10	6.8 %
	Defaulted	2	1.4 %
	Death	7	4.8 %
	Total	147	100 %

**Table 3:** Comparison of initial treatment outcomes of VL patients treated with low and high dose liposomal amphotericin B

Types of outcome	Outcomes	Low total dose liposomal amphotericin B (<24 mg/kg) (*N* = 86) *n* (%)	High total dose (24 -35 mg/kg) (*N* = 61) *n* (%)	*P* < 0.05
Initial treatment outcome	Cured	69 (80.2)	59 (96.7)	0.01
	Treatment failure	10 (11.62)	0	
	Defaulted	2(2.3)	0	
	Death	5(5.8)	2 (3.3)	

The median change in total white blood cell counts from baseline to end of treatment was not significantly (*p* = 0.7) different between the treatment groups (low and high dose liposomal amphotericin B). Similarly the median change in spleen size from baseline to end of treatment was not significant (*p* = 0.7) between the treatment groups. However, the median difference in platelet counts from baseline to end of treatment was significantly different between the treatment groups (*p* < 0.001), the count being higher in the high dose group [Table 4].

**Table 4:** Comparison of changes from baseline to end of treatment laboratory and clinical characteristics between the treatment groups

Variables	Low dose liposomal amphotericin B (<24 mg/kg total dose) *n* = 86	High dose liposomal amphotericin B (24-35 mg/kg total dose) *n* = 61	*P*-value
	Median (and IQR)	Median (and IQR)	
White blood cell (WBC) x 10^3^ cell/μl	1.65 (0.9 – 2.5)	1.7 (0.9 – 2.85)	0.7
Platelet x 10^3^ cell/μl	71.5 (37.8 – 142.5)	152 (92 – 238.5)	0.000
Spleen size in cm	4 (2 – 6.3)	4 (2 – 6.4)	0.7
Body temperature °C	2.3 (1.6 – 3.2)	2.2 (1.4 -3.2)	0.6

### Safety

Table 5 shows adverse events during treatment with liposomal amphotericine B. The commonest adverse events noted were hypokalemia in 39 (26.5 %) patients and infusion related reactions (mainly back pain, but also arthralgia and bone pain) in 16 (10.9 %). Other AEs were dyspepsia in 7(4.8 %), vomiting in 8 (5.4 %), elevation of liver transaminases in 7(4.8 %), and increased creatinine in 14(9.5 %). The difference in the occurrence of hypokalemia between the low [24 (28.2 %)] and high dose [15(24.2 %)] liposomal amphotericin B was not significant (*p* = 0.5). Similarly, the occurrence of infusion related reaction between the treatment groups was not significant (*p* = 0.2).

**Table 5:** Adverse events during treatment with liposomal amphotericin B

Adverse events (AEs)	Frequency	Percentage
No AE	56	38.1 %
Raised transaminase	7	4.8 %
Hypokalemia	39	26.5 %
Infusion related reaction	16	10.9 %
Dyspepsia	7	4.8 %
Vomiting	8	5.4 %
Increased serum creatinine from the baseline	14	9.5 %

## Discussion

The efficacy of anti-leishmanial drugs is known to vary from one geographic location to another [15, 16], even within the same continent, e.g., in Eastern Africa. Despite the fact that liposomal amphotericin B was introduced for the treatment of leishmaniasis in Ethiopia within the last decade, its effectiveness was not as high as seen in the Indian subcontinent [11]. While single dose liposomal amphotericin B was effective in India [17-19], recent studies have shown its ineffectiveness in Eastern Africa, especially in northwest Ethiopia and Sudan [20].

In this study, we show that the efficacy of liposomal amphotericin B in northwest Ethiopia was dose dependent, with higher efficacy at end of treatment (96.7 %) in high total doses of >24 mg/kg than lower total doses of <24 mg/kg [Table 3]. Due to the retrospective nature of this study, pharmacokinetic study was not conducted. Thus, we do not know if the serum level of the drug was varying with the doses used. Liposomal amphotericin B is used in a wide range of doses (3 – 5 mg/kg per dose) with varied recommendations of total dose for the different geographic areas. Generally higher total doses are being used in Mediterranean VL endemic areas. Of particular concern has been the absence of solid recommendations for VL in Eastern Africa. The appropriate dosage of the drug that produces higher efficacy and tolerability in Eastern Africa VL patients remains to be determined. Our data are indicative that higher doses are needed to effectively treat non-HIV VL patients in northwest Ethiopia. This is partly supported by a study which demonstrated failure of single dose AmBisome in Eastern Africa [20]. The geographical variation in the efficacy of AmBisome is intriguing and may be related to intrinsic variation in susceptibility of species and genotypes in the *L. donovani* complex even though host factors might well be contributing. To date, there is no definite proof of acquired resistance to the drug.

With respect to safety, hypokalemia [Table 5] is a major concern as was also observed in Asia [18]. It is therefore fair to recommend that hypokalemia has to be taken as a major adverse event related to use of liposomal amphotericin B that necessitates regular monitoring of serum potassium levels during treatment. The liposomal formulation of the drug needs cold chain during transportation and cold storage (<25 °C), but not freezing. Climatic conditions in most VL endemic regions are probably unsuitable to store at room temperature. Furthermore, routine treatment programs do not necessarily monitor temperatures. We are pointing to this issue as loss of the integrity of the liposomal formulation could possibly affect safety profile. In our setting, this is unlikely to be the case as the study site is a clinical trial site where standard operating procedures are in place for drug handling.

Based on assessment of initial treatment outcomes, our data suggest that liposomal amphotericin B could be an effective drug for the treatment of VL in East Africa if used at higher doses. Use of higher dosing will also have additional advantages in patients with concomitant fungal infections and neutropenia. On the other hand, higher doses incur high costs on the treatment. Dose finding studies are needed to find the appropriate high dose that is also safe.

The study has limitations in that more than 50 % of patients did not have a 6-month follow-up. Hence a critical assessment of final treatment outcomes could not be accomplished. We also discovered that due to unavailability of liposomal amphotericin B, a significant number of critically sick patients were treated with SSG. In Ethiopia, VL drugs are supplied by non-profit organizations e.g., the Drugs for Neglected Diseases initiative (DNDi) and Médecins Sans Frontière (MSF). The Federal Ministry of Health (FMOH) has in the recent years been distributing SSG and liposomal amphotericin B through its collaborative agreement with the World Health Organization (WHO). Liposomal amphotericin B is an essential addition to the package of anti-leishmanial drugs needed to treat VL in northwest Ethiopia, where patients present in various stages of severity and complications, including co-morbidities/co-infections, e.g., HIV and tuberculosis. Therefore, it is important that the supply is uninterrupted.

## Conclusion

In this retrospective study, we found that when liposomal amphotericin B was used as second-line treatment in complicated VL patients, initial treatment outcomes were dose dependent, and that higher doses (24-35 mg/kg) had better efficacy than lower doses (<24 mg/kg) in non-HIV VL patients from northwest Ethiopia. The appropriate efficacious and safe dose needs to be determined in well controlled clinical trials, and risk factors for the lower treatment outcomes need to be identified. Monitoring serum potassium level during treatment with liposomal amphotericin B should be an essential component of the clinical management of VL.

## Abbreviations

AEs, adverse events; BMI, body mass index; CAP, community acquired pneumonia; CI, confidence interval; DNDi, drugs for neglected disease initiative; FMOH, Federal Ministry of Health; GCP, good clinical practice; HIV, human immunodeficiency virus; IQR, interquartile range; LD, leishmania donovani; LRTC, Leishmaniasis Research and Treatment Center; MSF, Médecins Sans Frontière; OR, odds ratio; PTB, pulmonary tuberculosis; RDT, rapid diagnostic test; SD, standard deviation; SEM, standard error of mean; SPSS, statistical package for social science; SSG, sodium stibogluconate; ToC, test of cure; UoG, University of Gondar; VL, visceral leishmanaisis; WBC, white blood cell count; WHO, World Health Organization
